# Case Report: The importance of early intervention for gastroesophageal reflex disease caused by hiatal hernia

**DOI:** 10.3389/fped.2024.1305585

**Published:** 2024-05-13

**Authors:** Toshihiko Kakiuchi, Satoshi Obata, Azusa Koji, Nobuya Minematsu, Maho Fuchigami, Atsuhisa Fukuta, Tatsuro Tajiri, Masato Yoshiura

**Affiliations:** ^1^ Department of Pediatrics, Faculty of Medicine, Saga University, Saga, Japan; ^2^ Department of Pediatric Surgery, Graduate School of Medicine Sciences, Kyushu University, Fukuoka, Japan

**Keywords:** gastroesophageal reflux, hiatal hernia, child, fundoplication, proton pump inhibitor

## Abstract

**Background:**

Gastroesophageal reflux (GER) disease (GERD) is a condition wherein GER causes troublesome symptoms that can affect daily functioning and/or clinical complications within the esophagus or other systems. To avoid this, patients with GERD often require treatment; hence, it is important to distinguish GER from GERD. Patients with GERD exhibiting alarm signs should be examined early to differentiate it from GER and treated accordingly. Herein, we present a case of GERD caused by a hiatal hernia that required surgical intervention for esophagial cicatrical stenosis despite oral treatment. We also discussed how to choose the appropriate acid suppressants for GERD.

**Case presentation:**

A 1-year-old boy was referred to our hospital for repeated vomiting and poor weight gain. He received histamine 2 receptor antagonists (H2RAs) that contributed slightly to the decreased frequency of vomiting and aided weight gain; however, he soon stopped gaining weight and had bloody vomit. His upper gastrointestinal series revealed hiatal hernia, a 24 h impedance pH monitoring test indicated abnormal values for acid reflux, and esophagogastroduodenoscopy (EGD) revealed esophagitis. He was subsequently diagnosed with GERD associated with hiatal hernia. A proton pump inhibitor (PPI) was intravenously administered to him, following which his medication was changed to a potassium-competitive acid blocker (P-CAB). Thereafter, his vomiting episodes significantly decreased and his weight increased. However, 6 months after starting P-CAB, his vomiting episodes suddenly increased in frequency. EGD revealed the presence esophageal stricture due to scarring from GERD. He was then treated via laparoscopic fundoplication, gastrostomy, and esophageal balloon dilation. Thereafter, his vomiting episodes stopped and food intake improved, leading to weight gain.

**Conclusion:**

It is essential to identify the cause of GERD early and take an appropriate treatment approach depending on the cause of GERD with alarm signs. Further, as a drug therapy for GERD as a clear acid mediated disease or in children with alarm signs, PPIs or P-CAB should be used from the beginning instead of H2RAs.

## 1 Introduction

As per the combined guidelines of the European and the North American Societies for Pediatric Gastroenterology, Hepatology, and Nutrition (1), gastroesophageal reflux (GER) is defined as the passage of gastric contents into the esophagus with or without regurgitation along with vomiting. GER disease (GERD) is a condition that occurs when GER leads to troublesome symptoms that affect daily functioning and/or leads to clinical complications within the esophagus or other systems. GER is commonly observed in children, particularly in the first year of life. Up to 65% of infants aged 3–6 months regurgitate stomach contents at least once a day. This resolve spontaneously in most cases, with complete resolution reported in 95% of babies by 1 year of age (2). Conversely, the prevalence of GERD tends to decrease with time, from 25.5% at 1 month of age to 26.5% at 6 weeks, 7.7% at 3 months, 2.6%–2.9% at 6 months, and only 1.1%–1.6% at 12 months (3).

Considering that vomiting in babies is common, distinguishing GER from GERD may often be tricky, even for pediatricians (4, 5). Making this distinction between the two conditions is important because patients with GERD sometimes require treatment. Various factors including defects in the esophageal mucosal defense, impaired esophageal and gastric motility and clearance, and anatomical defects of the lower esophageal reflux barrier, such as hiatal hernia, play a role in the pathogenesis of GERD (3). The accurate distinction between these two entities is pivotal for the correct management of GERD as it will be the based for decisions about further investigation and treatment. Patients with GERD exhibiting alarm signs should be examined early and distinguished from GER. Regarding infants, most reflux episodes in infants are weakly acidic or nonacidic, indicating a significant unmet need for the management of infant GER(D) (6).

Herein, we present the case of a patient with GERD caused by a hiatal hernia with alarm signs (failure to thrive). The case required surgical intervention for esophageal cicatricial stenosis despite oral treatment.

## 2 Case description

A 1-year-old boy was referred to our hospital for repeated vomiting and poor weight gain. He was born full-term term, weighed 3,030 g at birth, and was delivered without asphyxia, but he vomited frequently after birth. His medical check-up at 1 month showed normal findings; however, his parents visited a pediatric clinic because of continued episodes of vomiting. Hypertrophic pyloric stenosis was ruled out by the pediatrician based on the symptoms, and it was considered that vomiting was accompanied by aerophagia. Non-pharmacology treatment, such as positioning therapy, parental guidance, education, and support, was provided to both the child and his parents. At 6 months of age, he was referred to the pediatrics department of a secondary hospital because of poor weight gain [5,490 g, −2.8 standard deviation (SD)]. He was in good general condition and was on breast milk, milk, and baby food. The frequency of vomiting was approximately five times a day, and defecation frequency was once a day. His blood tests, including endocrine function test, showed no abnormalities, and abdominal ultrasound, abdominal computed tomography (CT), and head magnetic resonance imaging scans showed normal findings. He was administered histamine 2 receptor antagonists (H2RAs) and Tsumura-Kampo Rikkunshito, a Japanese herbal medicine that has been attracting global attention and is under extensive research (7), both of which contributed slightly to decreasing the frequency of vomiting and aided in weight gain. However, he soon stopped gaining weight and presented with bloody vomiting; he was then referred to our hospital at 1 year of age.

**Figure 1:**
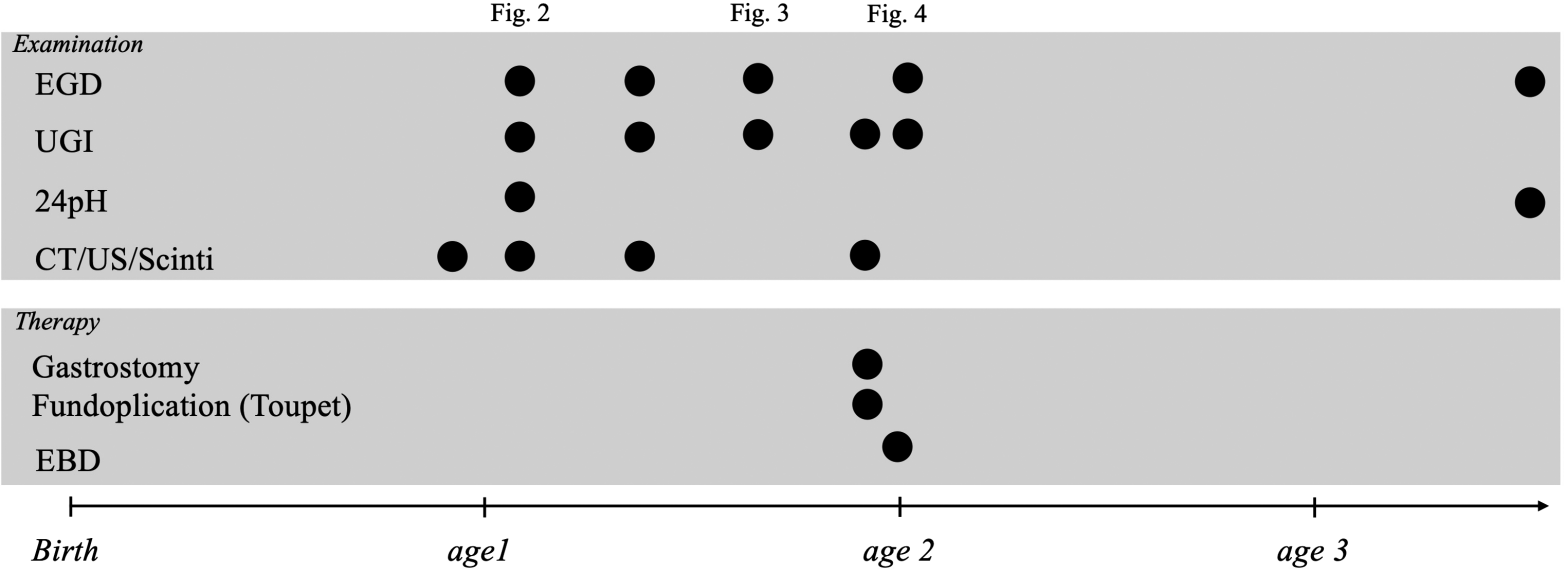
Patient's clinical course, including examinations and invasive treatments. EGD, esophagogastroduodenoscopy; UGI, upper gastrointestinal series; CT, computed tomography; US, ultrasonography, EBD, endoscopic balloon dilation.

**Figure 2:**
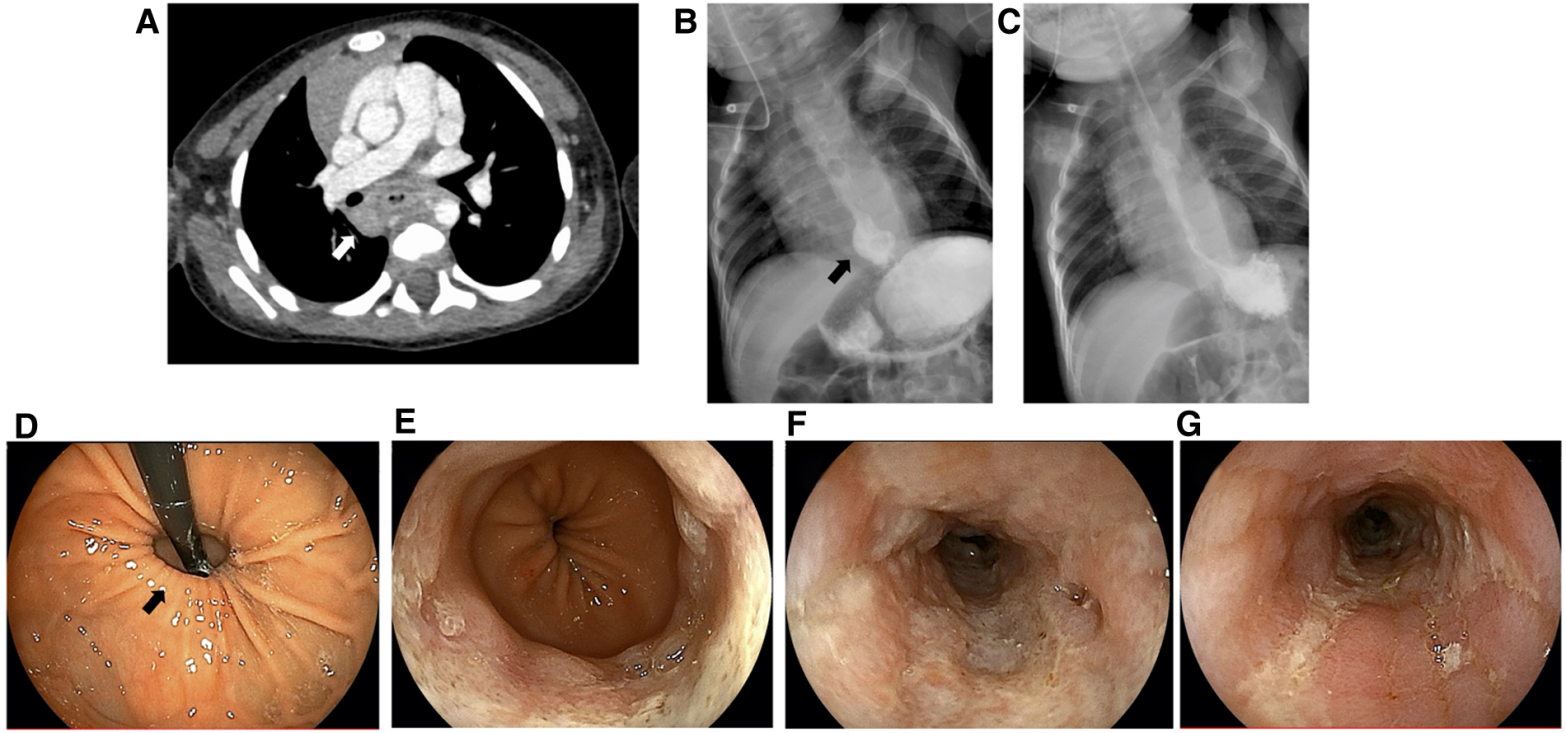
Abdominal computed tomography (**A**), upper gastrointestinal series (**B**,**C**), and esophagogastroduodenoscopy findings (**D**–**G**). White arrow, lymphadenopathy; black arrow, hiatal hernia.

At the time of his first visit to our hospital, his height was 70.4 cm (−1.8 SD) and his weight was 6.88 kg (−2.6 SD), indicating failure to thrive. Figure 1 presents his clinical course, including examinations and invasive treatments. Abdominal enhanced CT indicated no esophageal wall thickening, but it revealed the presence of enlarged lymph nodes around the esophagus (Figure 2A). His upper gastrointestinal (UGI) series revealed no obvious esophageal narrowing or dilatation but a hiatal hernia was noted (Figures 2B,C). No problems were observed in the outflow of the contrast medium from the esophagus to the stomach and duodenum, and the C-loop formation in the duodenum (shape from the descending limb of the duodenum to the Treitz ligament) was also normal. During UGI examination, reflux from the stomach to esophagus was observed. A 24 h impedance pH monitoring test showed abnormal values for both acid reflux (12.1%; normal range < 1.1%) and nonacid reflux (12.7%; normal range < 1.4%). Esophagogastroduodenoscopy (EGD) confirmed the hiatal hernia (Figures 2D,E) and also revealed severe mucosal erosion from the lower esophagus to the upper esophagus along with ulcer formation in some areas of the esophagus (Figures 2E–G). No obvious abnormality was observed from the stomach to the duodenum. The patient was then diagnosed with gastroesophageal reflux disease (GERD) associated with a hiatal hernia of the esophagus. He was administered a proton pump inhibitor (PPIs) via intravenous drip; thereafter, he was switched to a potassium-competitive acid blocker (P-CAB). After a confirmed decrease in vomiting episodes and increase in weight, he was discharged from our hospital on the 9th day after admission. His progress after discharge was fairly uneventful, and he gained some weight and only vomited once a day.

At 3 months after the initial hospitalization (i.e., at 1.5 years of age), the patient had one episode of defecation with black stool and his hemoglobin level dropped to 6.2 g/dl, indicating anemia; thus, he was readmitted to the hospital. UGI revealed no obvious esophageal narrowing or dilatation, but a hiatal hernia was noted again. Enhanced abdominal CT revealed thickening of the esophageal wall with enlarged lymph nodes around the esophagus, but these nodes were smaller than those observed in last examination. EGD revealed that the hiatal hernia had neither worsened nor improved. The erosion and ulcers in the esophagus began to improve, but scarring and subsequent pseudodiverticulum formation due to inflammation were noted. Several bleeding spots were observed on the mucosal surface within the hernia but without any active bleeding. Meckel's diverticulum scintigraphy was performed to look for the source of bleeding but no abnormality was noted. Red cell concentrate transfusion was conducted, following which it was confirmed that there was no progression of anemia. The patient was subsequently discharged from the hospital. Even after discharge, no black stool was observed, the frequency of vomiting decreased to approximately once a week, and the patient gained weight. It was concluded that this anemia was caused by gastrointestinal bleeding from within the hernia.

**Figure 3:**
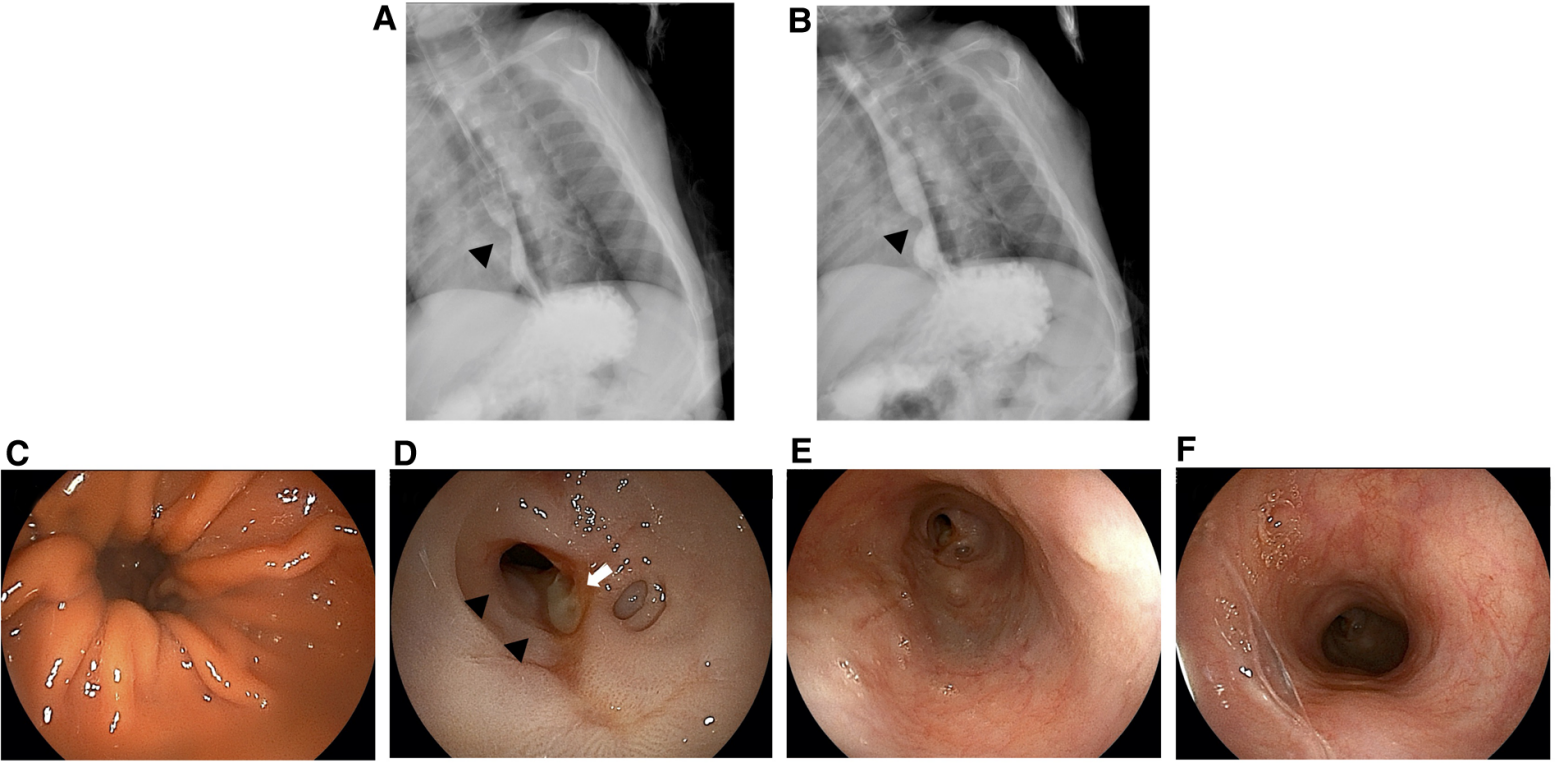
Upper gastrointestinal series (**A**,**B**) and esophagogastroduodenoscopy findings (**C**–**F**) at the age of 1 year and 9 months. White arrow, ulcer; black arrowheads, esophageal stricture.

At 3 months after the second hospitalization (i.e., at the age of 1 year and 9 months), the vomiting frequency of the patient suddenly increased from once a week to three times a day. Subsequently, P-CAB, sodium alginate, and ecabet sodium was reinitiated. UGI revealed obvious esophageal narrowing (Figures 3A,B) but EGD did not indicate any worsening of the hiatal hernia (Figure 3C). Moreover, ulcer formation was observed in the esophagus at the same location where scarring had been observed in the previous EGD (Figure 3D), and a stricture had appeared (Figures 3E,F). The cause of this recurrent episode of vomiting was considered to be esophageal stricture from the scarring from GERD caused by a hiatal hernia. In this case, medical treatment was considered to be ineffective, necessitating surgical intervention. Accordingly, the patient was referred to a pediatric surgery hospital.

**Figure 4:**
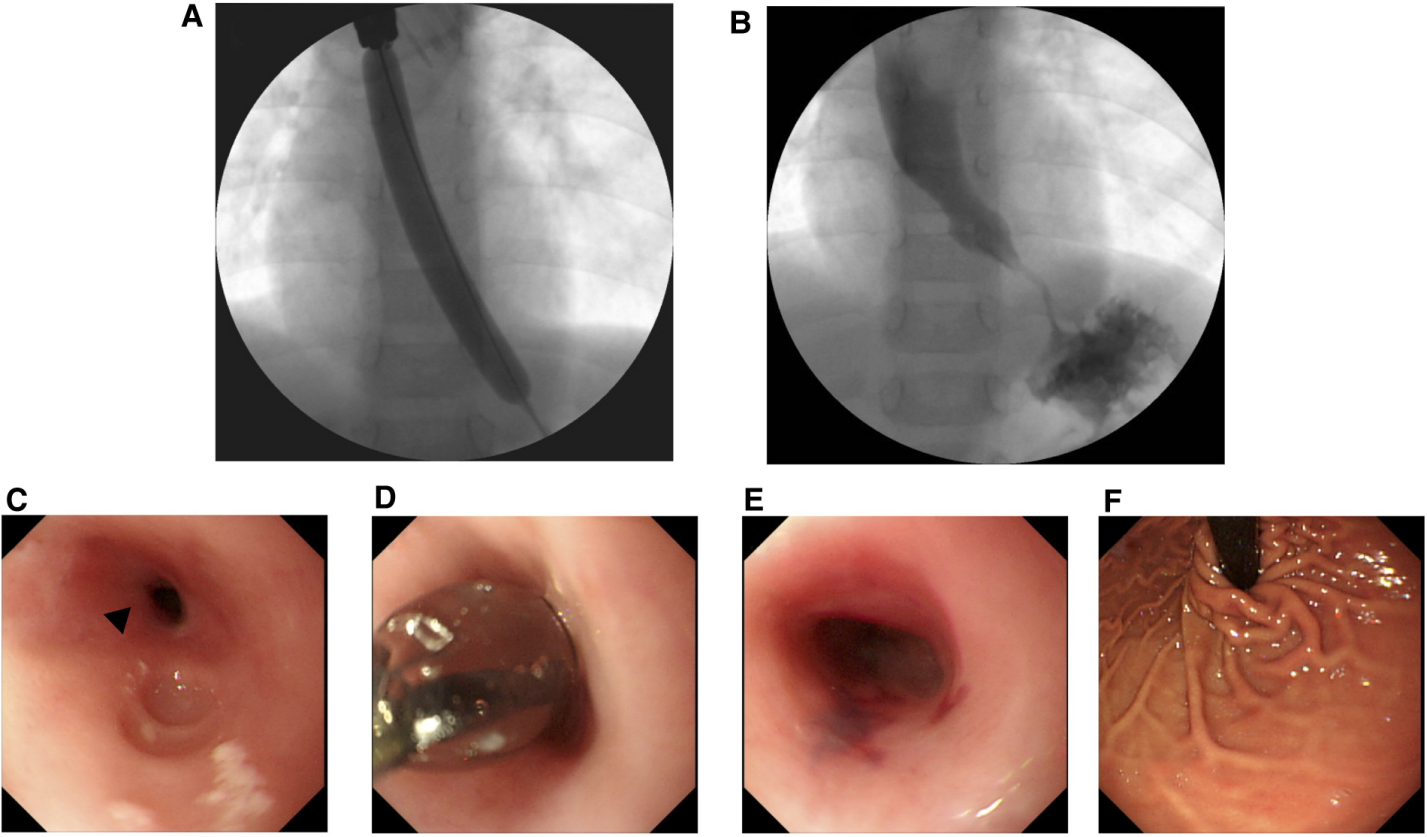
Upper gastrointestinal series (**A**,**B**) and esophagogastroduodenoscopy findings (**C**–**F**) during endoscopic balloon dilation. Black arrowheads, esophageal stricture.

Three months later (i.e., at 2 years of age), laparoscopic fundoplication using Toupet method (8) and gastrostomy were performed for the hiatal hernia at the surgery hospital. Further, 1 month later, esophageal balloon dilation was performed successfully under general anesthesia, and the esophageal stricture was successfully dilated (Figures 4A–E). Subsequent EGD revealed that the hiatal hernia had properly reduced (Figure 4F). Oral food intake was gradually started, and no vomiting was observed. UGI at 1.5 years after the surgery (at 3.5 years of age) showed no esophageal stricture. Moreover, no inflammation, scarring, stricture, or hiatal hernia of the esophagus was observed on EGD. Currently, the patient has reported no gastrostomy and vomiting, and his weight has returned to normal.

## 3 Discussion

The clinical course of this case highlights two important issues. First, it is necessary to identify the cause of GERD and take an early appropriate treatment approach depending on the cause of GERD with alarm signs (1). For this purpose, a broad differential diagnosis should be maintained and appropriate additional workup should be performed for patients with alarm signs and a clinical trajectory differing from that of standard infant reflux. Second, pharmacological therapy for GERD as a clear acid-mediated disease or in children with alarm signs should initially include PPIs or P-CAB rather than H2RAs.

In the present case, despite receiving oral H2RAs, the patient exhibited impaired growth and eventually bloody vomiting, and his digestive system was examined in detail. In the absence of alarm signs, patients should be treated conservatively by modifying feeds and posture. Poddar (9) reported that hematemesis, abnormal posturing, choking/gagging, or coughing while feeding, and regurgitation in infancy need not be investigated unless there are warning features, such as failure to thrive. Contrarily, if the patient presents with alarm symptoms, he should not be treated directly but instead should be examined to obtain a diagnosis (5). Therefore, GERD with alarm symptoms warrants immediate investigation to identify the cause and initiate necessary treatment. A key issue is distinguishing the clinical manifestations of GER with those of GERD in term infants, children, and adolescents to identify those who can be managed with pediatric conservative treatment and to refer those requiring pediatric gastroenterologist or pediatric surgeon consultation (10). This approach is suggested as there is no highly sensitive and specific and easily available bedside test for GERD (11).

A hiatus hernia impairs lower esophageal sphincter (LOS) competence, decreases LOS pressure and length, and alters the opening characteristics of the gastroesophageal junction, all of which can result in delayed clearance of and increased exposure to esophageal acid. Numerous studies have indicated that a hiatus hernia is associated with GERD symptoms, endoscopic esophagitis, Barrett's esophagus, and esophageal adenocarcinoma (12). The incidence of esophageal stricture is 1.1 per 10,000 person-years and increases markedly with age. The incidence of esophageal stricture decreased from 1994–2000, concomitant with a substantial increase in PPI usage, with most cases being of peptic strictures. Prior dysphagia, GERD, hiatus hernia, peptic ulcer disease, and heavy alcohol use are associated with an increased risk of esophageal stricture in adults (13). In contrast, data on pediatric cases is limited.

Furthermore, pharmacological therapy for GERD should initially include PPIs or P-CAB rather than H2RAs. In the present case, at 1 year and 2 months of age, the patient was diagnosed with GERD caused by a hiatal hernia. After using PPIs, the treatment was changed from H2RAs to P-CAB. Although GERD-associated vomiting temporarily improved after treatment, vomiting associated with esophageal cicatricial stenosis appeared approximately 6 months later. Although the possibility that this vomiting was a symptom associated with GERD could not be completely ruled out, EGD clearly revealed the narrowing of the esophagus (Figure 4). Esophageal cicatricial stenosis was probably the cause of the vomiting. PPIs and H2RAs are used as the gold standard of GERD treatment (1). However, PPIs are more effective for acid suppression than H2RAs (14), and there is no tachyphylaxis associated with prolonged used. However, this may not be effective in patients with nonacid or weakly acid reflux, and their prolonged use can also lead to increased respiratory rates and gastrointestinal infections (15, 16). H2RAs treatment have some limitations. Particularly, a fairly rapid tachyphylaxis can develop within 6 weeks of treatment initiation, limiting its potential for long-term use (17). Additionally, H2RAs are less effective than PPIs in terms of symptom relief and healing rates of erosive esophagitis. Although most of these limitations have been reported most clearly in adults, they are also believed to apply to children (10). Regarding esophagitis associated with GERD, the rate of complete cure with PPI treatment was greater than that with H2RA treatment. Thus, the likelihood of esophagitis healing is directly related to the potency of the antisecretory effect of the medication (18). Accordingly, stronger acid suppressors should be more effective in preventing esophageal strictures associated with GERD. P-CABs, such as vonoprazan (VPZ), are novel, potent acid-inhibitory drugs that competitively inhibit the binding of potassium to hydrogen-potassium ATPase in gastric parietal cells more efficiently than PPIs (19, 20). VPZ was made available in Japan in 2015 (21). P-CAB reportedly suppresses gastric acid production more strongly than PPIs, and by greatly suppressing esophageal inflammation in GERD, P-CABs can potentially inhibit the development of cicatricial stenosis more effectively than PPIs. However, limited studies have reported using P-CAB in children, and safety evaluations are insufficient. In the present case, with both acid and nonacid reflux, the clinical outcome may have improved and esophageal cicatricial stenosis could have been avoided if PPIs or P-CAB were used from the beginning rather than H2RAs.

In conclusion, it is necessary to identify early the cause of GERD and take an appropriate treatment approach depending on the cause of GERD with alarm signs. As a drug therapy for GERD as a clear acid mediated disease or in children with alarm signs, PPIs or P-CAB should be administered from the beginning instead of H2RAs.

## Data Availability

The original contributions presented in the study are included in the article/Supplementary Material, further inquiries can be directed to the corresponding author.
